# Intradural Extramedullary Hemangioblastoma of the Cervical Spine: Case Report and Literature Review

**DOI:** 10.7759/cureus.25125

**Published:** 2022-05-18

**Authors:** Andrew Fanous, Luke Mugge, Kelly Kurzejewski, April Cournoyer, Mateo Ziu

**Affiliations:** 1 Neurological Surgery, Inova Fairfax Hospital, Falls Church Virginia , USA; 2 Department of Neurosurgery, Inova Neuroscience and Spine Institute, Falls Church, USA; 3 Department of Neurosurgery, Inova Neuroscience and Spine Institute, Alexandria, USA; 4 Department of Neurosurgery, Inova Neuroscience and Spine Institute, Fairfax, USA

**Keywords:** spine, extramedullary, intradural, hemangioblastoma, von hippel-lindau

## Abstract

Hemangioblastomas are uncommon in the spine, accounting for less than 3% of all spinal cord tumors and occurring even more rarely in the intradural extramedullary vicinity. We present a unique case report of an intradural extramedullary hemangioblastoma. A 62-year-old man presented with a five-month history of neck pain radiating to the left arm. A magnetic resonance imaging (MRI) of the cervical spine revealed a left paracentral contrast-enhancing intradural extramedullary lesion at the C4-C5 level. Surgical options were discussed, and surgery was performed via a posterolateral approach. The lateral masses and facets at the C4 and C5 levels were drilled and the tumor was encountered ventral to the spinal cord. There were multiple nerve roots adherent to the tumor capsule. The tumor was highly vascularized. Analysis revealed a highly vascular lesion with vacuolated tumor cells, positive for inhibin and S100 stains, consistent with a diagnosis of hemangioblastoma. The patient remains intact throughout the post-operative period. Few studies have reported intradural extramedullary spinal hemangioblastomas and purely extramedullary spinal hemangioblastomas of the neuraxis are far less common. Most cases occur in the Japanese population and in patients over the age of 50. By location, extramedullary hemangioblastomas involving the thoracic spine occur in women, while those occurring in men are restricted to the cervical spine or conus medullaris. Complete resection remains the treatment of choice.

## Introduction

Hemangioblastomas are vascular tumors of the central nervous system that occur most commonly in the cerebellum, typically in association with von Hippel-Lindau (VHL) disease [1]. These tumors are uncommon in the spine, accounting for less than 3% of all spinal cord tumors [2]. When encountered in the spine, they are almost exclusively intramedullary. Very few case studies of intradural extramedullary hemangioblastomas have been reported in the literature. The purpose of the current report is to highlight this unusual diagnosis and discuss the demographic characteristics of patients with these rare tumors.

## Case presentation

History and presentation

**Figure 1:**
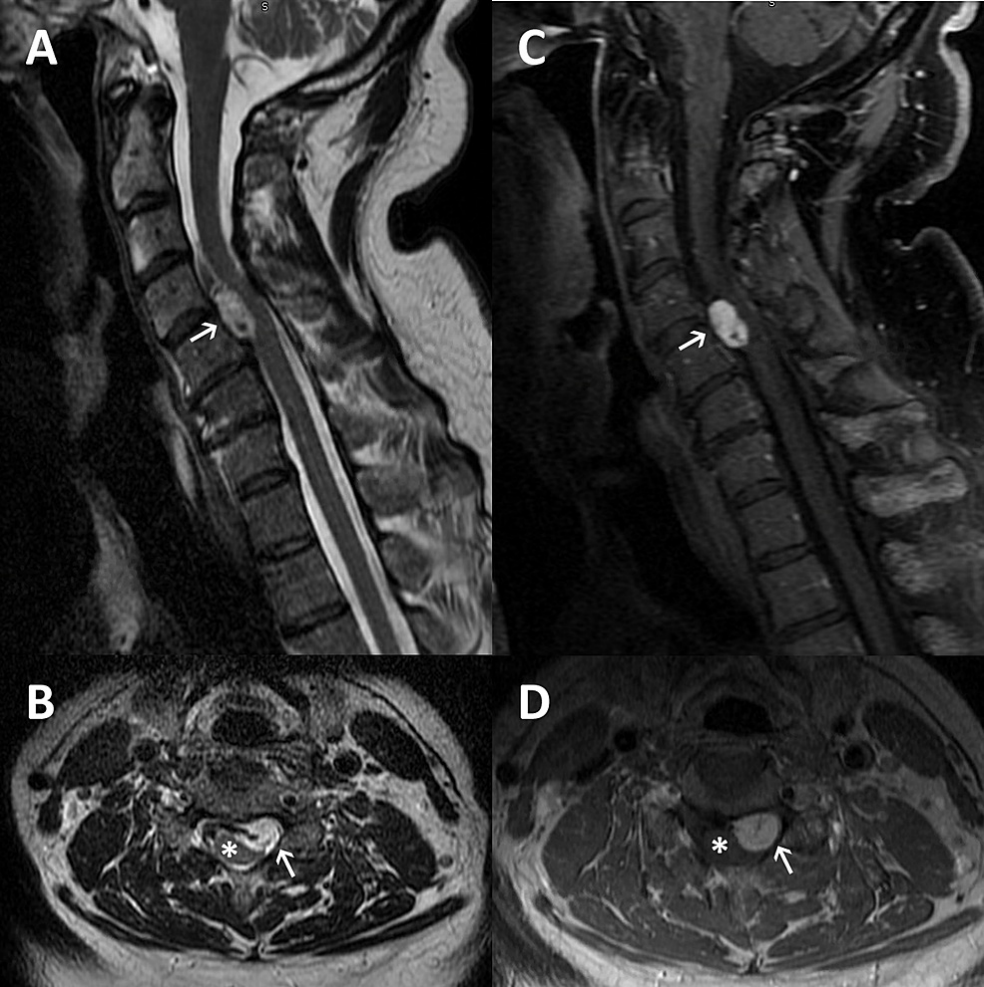
Pre-operative MRI MRI of the cervical spine demonstrating a left paracentral ventral intradural extramedullary lesion (white arrow) causing spinal cord (*) compression. (A) Sagittal view of a T2-weighted image; (B) axial view of a T2-weighted image; (C) sagittal view of a T1-weighted image with contrast administration; (D) axial view of a T1-weighted image with contrast administration.

A 62-year-old Caucasian male presented with a five-month history of neck pain radiating to the left arm. The patient had no history or stigmata of VHL disease. On physical examination, the patient was severely myelopathic. A magnetic resonance imaging (MRI) of the cervical spine revealed a left-paracentral contrast-enhancing intradural extramedullary lesion at the C4-C5 level, causing severe compression of the spinal cord (Figure 1). Given the patient’s examination and imaging findings, surgical resection of the lesion and decompression of the spinal cord was offered. After a complete discussion of surgical benefits and risks, the patient elected to proceed with surgical intervention.

Surgery

**Figure 2:**
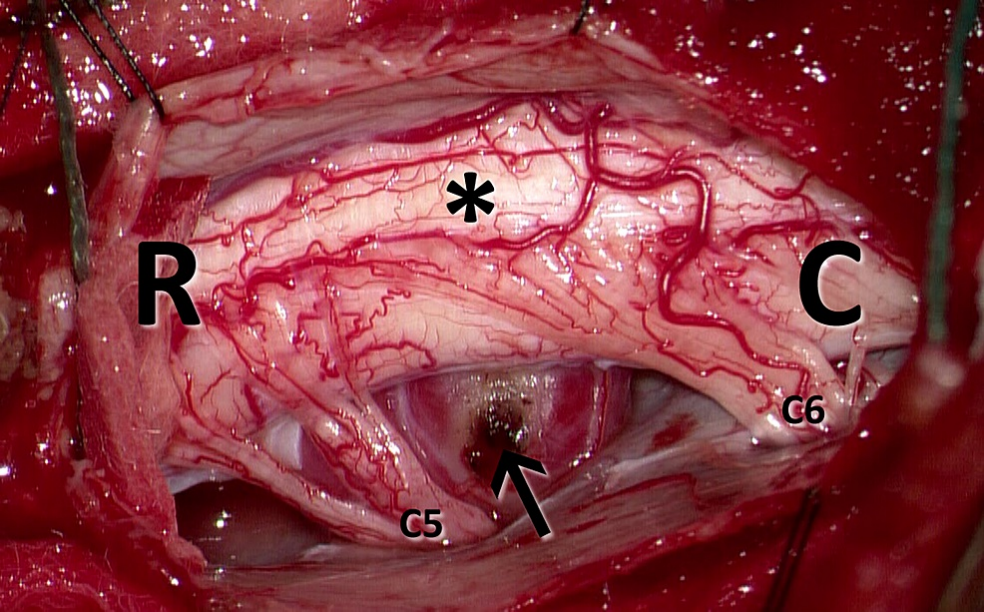
Intraoperative imaging Intraoperative image demonstrating a left paracentral intradural extramedullary lesion (black arrow) causing spinal cord (*) compression and dorsal displacement. [R] Rostral; [C] caudal; [C5] left C5 nerve root; [C6] left C6 nerve root.

Surgery was performed through a posterolateral approach. Instrumentation was moved from C3 to C6. No screw was placed on the left at the C4 level. After that, the lateral masses and facets at the C4 and C5 levels were drilled to permit access to the tumor. The tumor was encountered ventral to the spinal cord and was severely compressing and displacing it dorsally (Figure 2). There were multiple nerve roots adherent to the tumor capsule but not incorporated within it. The tumor was dark red in color and highly vascularized. Using microsurgical techniques, the nerve roots were carefully dissected free from the capsule. The tumor was subsequently debulked from within, and the capsule was resected, leading to complete decompression of the spinal cord. A water-tight closure of the dura was performed. Neuromonitoring signals remained unchanged throughout the procedure.

Pathological analysis

**Figure 3:**
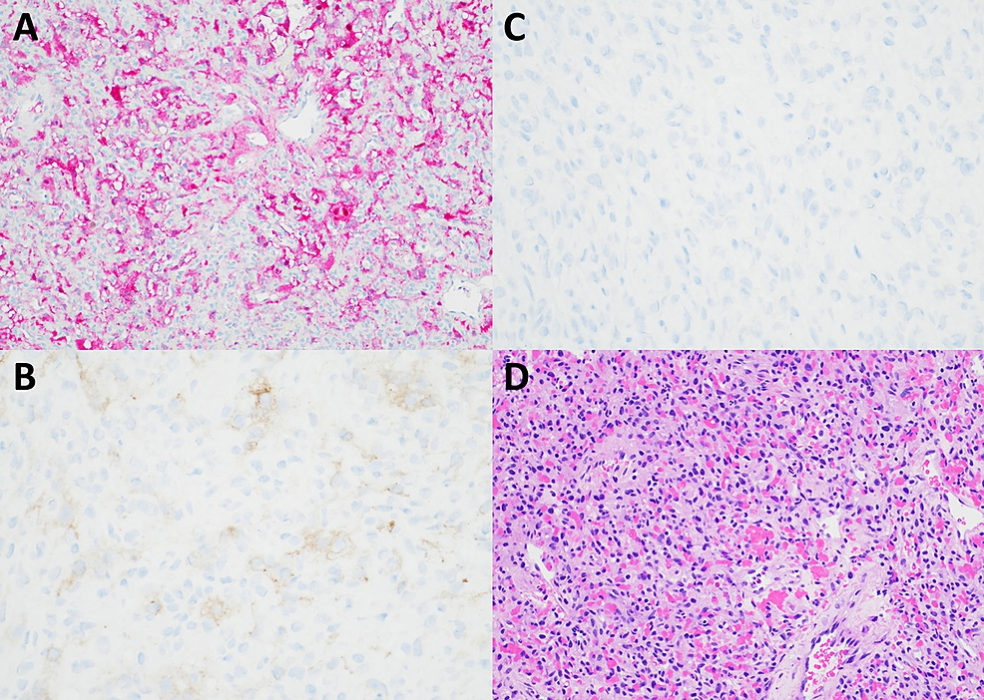
Histology Histopathological samples of the tumor demonstrated strong positivity for S100 (A) and inhibin (B) stains. EMA stain (C) was negative. (D) Hematoxylin–eosin stain demonstrates dense networks of vascular channels, as well as cells with nuclear pleomorphism. The final diagnosis is hemangioblastoma.

Pathological analysis of the specimen revealed a highly vascular lesion with vacuolated tumor cells (Figure 3). The cells were positive for inhibin and S100 stains. The epithelial membrane antigen (EMA) stain was negative. Given the morphologic and immunohistochemical features of the lesion, the final diagnosis was most consistent with hemangioblastoma.

Postoperative course and follow-up

**Figure 4:**
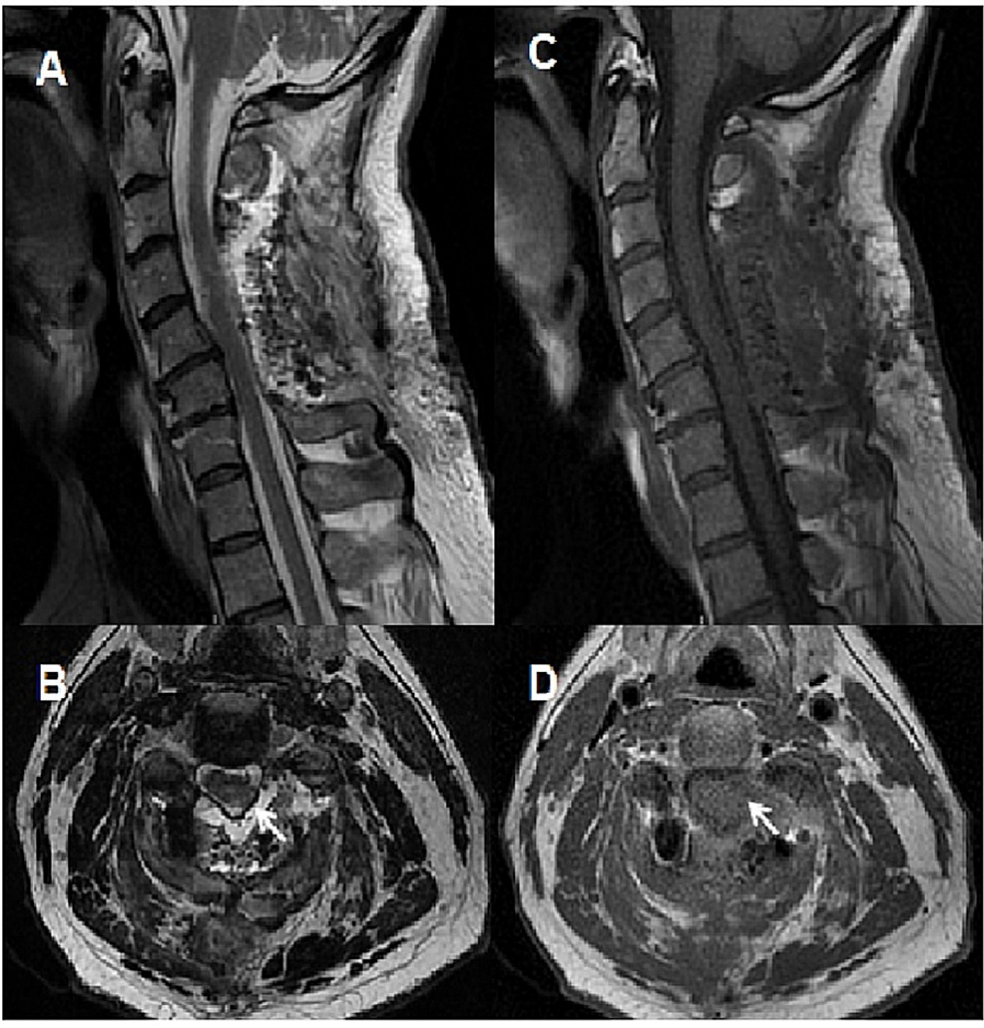
Post operative MRI of the cervical spine Post-operative MRI of the cervical spine demonstrating completed resection of the tumor with a cavity (white arrow). (A) Sagittal view of a T2-weighted image; (B) axial view of a T2-weighted image; (C) sagittal view of a T1-weighted image; (D) axial view of a T1-weighted image.

The patient’s myelopathy improved in the immediate postoperative period, and his strength and sensation remained intact. Postoperative MRI revealed complete resection of the tumor with no residual enhancement (Figure 4). At his one-month follow-up appointment, the patient had no pain and was not taking any pain medications. He was intact on neurological examination and had no evidence of myelopathy.

## Discussion

A few studies have reported intradural extramedullary spinal hemangioblastomas. Most of these cases were found in the Asian population, either in Japan or China. In an analysis of 116 cases of spinal hemangioblastomas, Deng et al. encountered 25 (22%) to be primarily extramedullary, defined as >50% of the tumor located in the extramedullary compartment [3]. This combined intramedullary-extramedullary growth of hemangioblastomas is not entirely uncommon. In a study of 85 cases of hemangioblastomas by Browne et al., 18 (21%) exhibited such exophytic growth [4]. Similarly, Yasargil et al. noted that 25% of resected spinal hemangioblastomas had a combined intramedullary-extramedullary component [5]. Furthermore, hemangioblastomas involving the filum terminale and cauda equina are rare [6].

However, purely extramedullary spinal hemangioblastomas of the neuraxis are far less common. In a study by Imagama et al. of spinal hemangioblastomas examining 26 patients, only one case was purely extramedullary [7]. In three separate reports, Kitanaka et al., Kawanishi et al., and Taniguchi et al. published cases of middle-aged Japanese women with intradural extramedullary hemangioblastomas located in the thoracic spine [2,8,9]. In two additional studies, Toyoda et al. and Minami et al. reported two cases of middle-aged Japanese men with cervical extramedullary hemangioblastomas [1,10]. Yasuda et al. studied 11 Japanese patients with spinal hemangioblastomas and found two cases of extramedullary tumors involving the thoracic spine and the conus medullaris [11].

In the non-Japanese population, the presence of extramedullary spinal hemangioblastomas is exceedingly rare. In a review of 85 cases of spinal hemangioblastomas, Browne et al. found only one case of extramedullary hemangioblastoma [4,9]. Wisoff et al. and Li et al. reported two cases of men with hemangioblastomas involving the cervical spine and conus medullaris, respectively [6,12,13]. In addition, Barbosa-Silva et al. and Brisman et al. reported two cases of such tumors occurring in the thoracic spine of two middle-aged women [14,15].

The demographic details of all patients found in the literature with purely intradural extramedullary hemangioblastoma can be found in Table 1. Most patients (57%) were Japanese. All the other reports originated in the United States, with the exception of one report from Brazil. Half of the patients were women, and the sex was not reported in one case. The average age for all patients was 59.5 years (a range of 46-81 years). The average age for women was 61.9 years (a range of 47-81 years) and the average age for men was 56.8 years (a range of 46-72 years). Age was not reported in one male patient. Interestingly, all extramedullary hemangioblastomas involving the thoracic spine occurred in women, while all those in men occurred in either the cervical spine or the conus medullaris.

**Table 1:** Intradural extramedullary hemangioblastoma List of all patients with purely intradural extramedullary hemangioblastoma found in the literature. F: female; M: male.

Case No.	Author	Year	Sex	Age	Location	Paper origin
1	Browne et al. [4]	1976	F	47	Thoracic	United States
2	Wisoff et al. [12]	1978	M	59	Conus	United States
3	Kitanaka et al. [9]	1993	F	59	Thoracic	Japan
4	Minami et al. [10]	1998	M	48	Cervical	Japan
5	Brisman et al. [15]	2000	F	57	Thoracic	United States
6	Toyoda et al. [1]	2004	M	46	Cervical	Japan
7	Taniguchi et al. [8]	2009	F	65	Thoracic	Japan
8	Barbosa-Silva et al. [14]	2009	F	66	Thoracic	Brazil
9	Imagama et al. [7]	2011	Not reported	Not reported	Thoracic	Japan
10	Yasuda et al. [11]	2016	M	54	Conus	Japan
11	Yasuda et al. [11]	2016	F	81	Thoracic	Japan
12	Li et al. [13]	2021	M	72	Cervical	United States
13	Kawanishi et al. [2]	2021	F	58	Thoracic	Japan
14	Fanous (current)	2022	M	62	Cervical	United States

Extramedullary hemangioblastomas are associated with smaller syrinxes than their intramedullary counterparts [11]. Since spinal meningiomas and schwannomas are rarely associated with syrinxes, the presence of a syrinx in connection with an extramedullary lesion should raise the suspicion of an extramedullary hemangioblastoma [2]. Other characteristics key to preoperative diagnosis includes the presence of marked contrast enhancement and associated enlarged vessels on MRI [14]. Complete surgical resection of these lesions is the treatment of choice since the residual tumor has been shown to regrow, conferring a poor prognosis [3].

## Conclusions

Hemangioblastomas within the spine are uncommon, but lesions located purely intradural but extramedullary are rarer still. While usually occurring within the context of VHL, these lesions can occur sporadically. Our patient did not have VHL, making this lesion unusual and unexpected. Additionally, our report contrasts with established literature, which suggests that these lesions predominantly occur within Asian populations. Surgery remains the gold standard of treatment. The accurate pathological analysis is critical given that postoperative care, including genetic analysis, is necessary.
